# The alternate-form reliability study of six variants of the Brief Visual-Spatial Memory Test-Revised and the Hopkins Verbal Learning Test-Revised

**DOI:** 10.3389/fpubh.2023.1096397

**Published:** 2023-03-22

**Authors:** Yumei Cai, Tianlong Yang, Xin Yu, Xue Han, Gong Chen, Chuan Shi

**Affiliations:** ^1^Peking University Institute of Population Research, Beijing, China; ^2^Anjia Hospital, Beijing, China; ^3^Peking University Institute of Mental Health (Sixth Hospital), Beijing, China; ^4^National Clinical Research Center for Mental Disorders, Peking University Sixth Hospital, Beijing, China; ^5^Key Laboratory of Mental Health, Ministry of Health, Peking University, Beijing, China

**Keywords:** practice effect, Brief Visual-Spatial Memory Test-Revised, Hopkins Verbal Learning Test-Revised, alternate-form reliability, cognitive assessment

## Abstract

**Introduction:**

The revised Hopkins Verbal Learning Test-Revised (HVLT-R) and the Brief Visual-Spatial Memory Test-Revised (BVMT-R) are two widely used test involving verbal and visual learning and memory. In the two tests, six different versions are assembled, respectively, to prevent learning effects. Currently, no researchers have compared the six versions of the two tests. Thus, their usefulness in clinical studies requiring multiple follow-ups is limited. In this work, we confirm the equivalence of six HVLT-R and BVMT-R versions.

**Methods:**

20 people completed all six HVLT-R and BVMT-R versions, while 120 people were randomly assigned to complete one of the six versions of each test. The Intelligence Quotient (IQ) level is measured using the short version of the Wechsler Adult Intelligence test. R4.2.0 is used for statistical analysis. The K-Related sample test (a non-parametric test) is used to observe the differences in test scores among the 20 subjects. The one-way Analysis of Variance (ANOVA) test is utilized to analyze the differences in test scores among the 120 subjects. The scores on different versions are compared using two similar sample tests. The HVLT-R Total Learning, the HVLT-R Delayed Recall, the BVMT-R Total Learning, and the BVMT-R Delayed Recall are indexes for comparison. Version and test scores are used as research factors, while different versions are used as research levels.

**Results:**

The results suggest that HVLT-R and BVMT-R versions 3, 5 and 6 are equally difficult, and relatively easy compared to versions 1, 2 and 4. HVLT-R versions 3, 5, and 6 show good reliability and can be used interchangeably when testing word learning ability or short-term memory; BVMT-R Versions 3, 5, and 6 show acceptable reliability and can be can be used interchangeably.

**Discussion:**

In the study of multiple follow-ups, it is a must to avoid discrepant versions and choose other equivalent versions. The results from this study could be used as a guide for upcoming studies and clinical applications in China.

## 1. Introduction

Neurocognitive assessment facilitates early detection of neurocognitive disorder. A fundamental constraint of neurocognitive assessment at the beginning of this century is the lack of consensus on how to evaluate cognition, including in specific cognitive tests and the field of cognitive assessment as a whole. The lack of consistent evaluation makes assessment and diagnosis complicated. This is a serious impediment to treatment, particularly for clinical trials and the use of cognitive drugs. In 2004, the National Institute of Mental Health (NIMH) initiated the MATRICS Consensus Cognitive Battery (MCCB) program, which included a series of consensus meetings with experts from across the country and proposed seven key fields of cognitive disorder in the disease. These key fields are believed to be the most damaged and relevant to the final outcome. They are working memory, attention/vigilance, language acquisition and memory, visual acquisition and memory, reasoning and problem-solving, processing speed, and social cognition (1). Its paramount components include language learning and memory tests in addition to visual learning and memory tests. Impairment in the field of learning and memory is the most common and prominent problem in cross-disease diagnosis. Consequently, the corresponding tests have also undergone substantial development.

Vocabulary Memory is a relatively common measure of language learning and memory used in clinical and research settings (2). There are several standard language learning and memory tests, such as Rey Auditory Verbal Learning Test (RAVLT) and California Verbal Learning Test (CVLT). Despite the fact that these tests have proven valuable in clinical and research settings, they had limitations. Its operation is difficult for patients with severe cognitive impairment. The short, easy-to-use design of HVLT-R was developed. Compared with RAVLT and CVLT, HVLT reduces the types of semantic categories, the number of words and the frequency of recall. Compared with RAVLT and CVLT, HVLT has optimized test structure. HVLT separated the semantically related words from the immediate recall and delayed recall tests and performed them separately in the recognition test. HVLT-R has been shown to evaluate patients with Alzheimer's disease, vascular dementia, and mild cognitive impairment (3).

At the same time, Visuospatial Memory has made great progress in clinical and scientific research. An increasing number of individuals support the use of visual memory tests in the diagnosis of dementia. The visual memory test has been identified as one of the most accurate predictors of the functional outcome of Alzheimer's disease dementia and has been shown to have a higher diagnostic value (4–8).

BVMT-R focuses on assessing cognitive processing speed and verbal and visual memory (9). Several studies, including the assessment of depression (10), multiple sclerosis (11), schizophrenia (12) and bipolar disorder (13) recommend BVMT-R for visual learning and memory assessment. There is evidence that the delayed recall and retention percentage score of BVMT-R can distinguish cognitive impairment between AD patients and those with Lewy bodies dementia, thereby improving the accuracy of diagnosis (14). There is also some support for its application to the assessment of cognitive deficits in Parkinson Dementia (15).

HVLT-R and BVMT-R are designed to be relatively short, easy to operate, and have the same form, so they are often used together. Shi et al. established the Chinese norm of MCCB (16). HVLT-R and BVMT-R have been clinically used in Chinese population. The validity of the tool was tested in schizophrenic patients, which made it a remarkable method for assessing neurocognitive disorder (17).

Chen et al. (18) used the MCCB tool to evaluate the differences in cognitive performance in patients with schizophrenia at different stages. They once again confirmed that cognitive disorder is the core symptom of schizophrenia. The initial phase of a disease is the most indispensable treatment phase all throughout entire course. It is suggested that attention should be paid to the neurocognitive changes of the first-episode patients. Constructive suggestions are given for the early intervention of neurocognitive disorder (18). Zhang et al. (19) conducted a meta-analysis of MCCB tools. Compared with healthy controls, the comprehensive MCCB score and each of the seven cognitive domains in Chinese schizophrenics both demonstrated significant deficiencies. Processing speed and attention had the biggest overall effects, followed by visual learning, working memory, language learning, problem-solving, and social cognition. The effect values of the seven cognitive domains ranged between −0.87 and −1.41. Social cognition shows the least damaged. Some subtests, such as symbol coding, the connection test, and the continuous attention test, are sensitive, which will be useful for the future development of cognitive batteries. In the field of depression, Liang et al. evaluated the psychological properties of the tool in depressed patients and confirmed the brilliant internal consistency and reliability of the MCCB in Chinese patients with MDD (20). In order to verify the psychometric characteristics of MCCB in adolescent patients with MDD, further research was carried out. The conclusion was drawn that MCCB shows excellent psychometric characteristics in adolescent MDD patients (21).

Shi et al. used the first version of two tests in the Chinese norm. The revised Hopkins memory test (HVLT-R) is a word list learning and memory test. It is mainly used for people with neurocognitive disorder. The HVLT-R test consisted of three learning tests, including 12 semantically classified words, followed by a 20-min delayed recall test, ending with a yes/no recognition test. The highest total learning score was 36 and the highest total delayed recall score was 12 (22). The original version of HVLT has been examined in pieces of literature (23). The convergence validity of the two methods was compared. For example, the analysis of standard HVLT and CVLT in a sample of healthy elderly people showed a good correlation between the measurement methods of total word learning (*r* = 0.74, *P* = 0.001). However, no consistent relationship was found between insertion or persistent errors in different tasks. These results support HVLT as a measure of learning ability. It also demonstrated the utility of immediate recall in normal elderly individuals. The empirical validity of HVLT-R has also been demonstrated in the studies of ordinary people and neuropsychiatric patients. Construct validity, criterion validity, and discriminant validity of the HVLT-R have been established in two populations with or without neurological disorder (3, 24, 25). The Brief Visual-spatial Memory Test (BVMT-R) is a visual graphic test tool developed by Benedict in recent years. In the current revised BVMT-R, 6 simple graphics (arranged in a 2-by-3 matrix) were visually presented to the subjects in the pamphlet. Three consecutive 10-s experiments were conducted. At the conclusion of each test, the participants must correctly draw as many patterns as possible. After a 25-min delay, they were again asked to draw the exact layout. The recognition test was conducted immediately after the delayed memory test. Recognition and recall were based on the accuracy of immediate recall and recall. For each graph, one for the correct position and one for the correct figure, with a maximum score of 12 per test (26). Due to the similar procedures of the HVLT-R test (e.g., 3 learning tests, 20–25 min delayed recall test, recognition test), there are six alternative forms, and these memory tests are relatively short, which are also suitable for patients with severe disorder. As a result, it is commonly utilized with the HVLT-R test. Among all cognitive tests, the learning and memory tests are the most likely to demonstrate practice effect. They are something we need to pay attention to in our clinical evaluation.

## 2. Materials and methods

### 2.1. Research subjects and their inclusion and exclusion criteria

The research subjects were community health ones who were included through recommendation and recruitment information from September 2020 to March 2021. The specific inclusion and exclusion criteria are as follows:

The inclusion criteria of healthy subjects are as follows:

(1) The subjects are between 18 and 65 years old.
(2) The subjects' Wechsler Adult Intelligence Scale−3rd Edition (WAIS-III) scores are >80.
(3) The subjects have signed informed consent.

The exclusion criteria of healthy subjects are as follows:

(1) The subjects currently or previously had any of the following diagnoses:
  a. Alcohol and/or substance use disorders,
  b. Autism spectrum disorder,
  c. Bipolar disorder,
  d. Dementia or any other neurodegenerative disease,
  e. Learning disabilities,
  f. Depressive disorder,
  g. Schizophrenia or other mental disorders,
  h. Other medical conditions that may affect cognitive function (such as brain tumor, multiple sclerosis, Parkinson's disease, etc.).
(2) The subjects had unstable medical diseases.
(3) The subjects were taking drugs that might affect cognitive function (e.g., glucocorticoidsβ-Receptor blockers, opioid analgesics, central stimulants, etc.).
(4) Subjects consumed alcohol within 8 h of implementation of BVMT-R and HLVT-R tools.
(5) The subjects could not read and understand the informed consent form or self-report questionnaire.

### 2.2. Research tools

#### 2.2.1. General survey

General demographic data: name, gender, age, education, marriage, residence, nationality, smoking, drinking, family history, etc.

#### 2.2.2. Intelligence assessment

Gong and Dai created the Wechsler Adult Intelligence Scale-−3rd Edition (WAIS-III) in 1984 (27). It has been used to assess the overall intelligence level of individuals over the age of 16 in a relatively short period of time. This study primarily assesses knowledge span, learning and acceptance ability, material memory ability, and ability to recognize everyday things, with a maximum original score of 29. The arithmetic test mainly assesses the reasoning ability and active attention ability of mathematical calculation, with a maximum original score of 18. The similarity test mainly assesses logical thinking ability, abstract thinking ability, generalization ability, with a maximum original score of 26. The digital span test mainly assesses attention and short-term memory ability (including forward and backward numbers), with a maximum original score of 22. Then, the four subtests' initial scores are translated into the coarse subscale score in accordance with the age range. In order to calculate the overall scale score, the coarse scores from the four subtests are added, divided by 4, multiplied by 11, and finally converted to an age-appropriate IQ value.

### 2.3. Study design

#### 2.3.1. HVLT-R memory test evaluation

In the HVLT-R test, subjects were asked to speak at a strict two-second rate of one word. In addition, during the operation, we should truthfully record the words answered by the subjects. Instead of asking subjects to put a check mark after the corresponding word. This is because the semantically related approximate answers can reflect the level of semantic memory ability of the subjects, which is convenient for us to analyze after the test. Throughout the test, we did not give any indication of what was right or wrong.

Step 1: Say to the subjects, “next, I'm going to read you a set of words. Please listen carefully, because when I finish reading, I want you to say as many words as you can remember. You can say them in any order. Are you ready?” Read the list at the rate of one word every 2 s. If the subject doesn't automatically start to report the word after the last word is read, say, “OK, now please tell me as many words as you can remember.” You can gently and quickly ask the subjects if they can remember the rest of the words: “Can you remember more?”
Step 2: When the subjects say they can't think of more words, say, “now let's do it again. I'll read you the same set of words. Please listen carefully and say as many words as you can remember. You can say them in any order, including the words you told me for the first time.” Read the list at the rate of one word every 2 s.
Step 3: When the subjects say they can't think of more words, they say, “I'll read this group of words again. Like just now, I want you to say as many words as you can remember. You can say them in any order, including the words you told me for the first time.” When the subjects indicated that they could not think of more words, then record the time in the completion time column of the 3-step test. The delayed test will be done in the next 20 min.

#### 2.3.2. BVMT-R memory test evaluation

BVMT-R Test 1 measures short-term visual memory and attention. Tests 2 and 3 measure learning and long-term visual memory. Therefore, the test was conducted strictly according to the exposure time of the stimulus of 10 s. Delayed recall measures long-term visuospatial memory skills and the ability to retrieve information from long-term memory. The total recall score reflects the overall level of visual memory.

In front of the subjects, place a response sheet and a pencil with an eraser. Before the start of each learning experiment, the subjects' attention should be focused on the manual containing the recall stimulus.

Step 1: I'm going to show you a card with six graphics. I want you to learn these graphics and remember them as much as possible. You only have 10 s to learn the whole list. I'm going to show them here. After that, I put the card about 40 cm away from the subject's eyes, and try to draw every figure exactly where it appears.
Step 2: Good, I'd like to see if you can remember more graphics if you have another chance. I'll show you another 10 s, and this time try to remember as many of these figures as possible, including the last one, and try to draw each figure accurately and put them in the right place.
Step 3: If you get a second chance, I'd like to test your memory by asking you to recall more graphics. I'll demonstrate for another 10 s. This time, try to recall as many of these figures as you can, including the final one. Also, make sure that you accurately depict each figure and place it in its proper location.

The time was recorded in the completion time column of the 3-step test when the subject indicated he or she could not generate any additional graphs. The delayed test will be completed in 25 min.

#### 2.3.3. Sample size estimation

The sample size of healthy subjects refers to the number of subjects included in the test of replica reliability in the HVLT-R and BVMT-R manuals (28, 29). In this work, 20 subjects are recruited to complete all 6 tests of HVLT-R and BVMT-R. The remaining 120 subjects are assigned one of the 6 sets of HVLT-R and BVMT-R by random program numbering method. The 120 and 20 subjects were all from the community and participated voluntarily. All the subjects were community residents from all over the country, but living in Beijing. The subjects' jobs were in all walks of life. Among them, 20 subjects were administered a different version of each test every other week.

### 2.4. Data management and analysis

#### 2.4.1. Data entry and management

Using R4.2.0 to input data, all subjects' general demographic data and cognitive test data are input into the database by two researchers for double entry verification and correction.

#### 2.4.2. Statistical analysis

R4.2.0 is used for statistical analysis. The general data includes age and education level (year), which are continuous variables, thus described by means and standard deviation. Their differences are compared by one-way ANOVA. Gender is a categorical variable, and the χ2 test is used to compare the difference.

Main outcome measures: HVLT-R Total Learning, HVLT-R Delayed Recall, BVMT-R Total Learning, BVMT-R Delayed Recall Total Learning = Trial 1 + Trial 2 + Trial 3. The presented results are rough scores. Version and test scores are used as research factors and different versions are taken as research levels.

Reliability: copy reliability. The difference of test scores between versions of 20 subjects is statistically tested by K-Related sample test in nonparametric test. And the difference of test scores between versions of 120 subjects is tested by one-way ANOVA. Two versions of the test are compared.

## 3. Results

### 3.1. General data analysis

According to the proportion of national census data in 2017 (gender: Men 51%, Women 49%), (education level: 39% of junior high school or below, 24% of senior high school, 37% of junior college or above), and (age: 30.1% of 20–35 years old, 25.6% of 36–50 years old, 20.6% of 50–65 years old), 124 healthy volunteers (subjects) are selected voluntarily. Through interview and test, 4 subjects received scores of < 80 in the third edition of the Wechsler Adult Intelligence Test and are excluded. For the remaining 120 subjects, one of the 6 sets of HVLT-R and BVMT-R is chosen by random program numbering. BVMT-R and HVLT-R tests are completed on 120 subjects and analyzed. Among the 120 subjects, the distribution of age, education (years) and gender are similar (Table 1). Among the subjects, 104 are smokers, 16 are non-smokers, 86 are non-drinkers, 25 are occasional drinkers, 9 are non-drinkers, 30 are unmarried, 83 are married, 2 are widowed, and 5 are divorced. Twenty additional individuals who completed all six HVLT-R and BVMT-R tests were included in the analysis. The degree of education (years) follows normal distribution, while the gender and age are not.

**Table 1:** Comparison of general data of 120 healthy subjects.

**Item**		**Age**	**Education level (year)**	**Gender** ***n*** **(%)**	
				**Men**	**Women**
Form1	*M*	40.95	12.95	11 (55%)	9 (45%)
	*SD*	11.62	3.35		
Form2	*M*	43.75	11.85	10 (50%)	10 (50%)
	*SD*	12.45	2.87		
Form3	*M*	38.40	12.60	7 (35%)	13 (65%)
	*SD*	13.63	3.55		
Form4	*M*	39.60	13.75	11 (55%)	9 (45%)
	*SD*	9.70	3.64		
Form5	*M*	40.80	12.35	12 (60%)	8 (40%)
	*SD*	9.69	3.44		
Form6	*M*	38.35	10.65	11 (55%)	9 (45%)
	*SD*	14.07	2.11		
F/χ^2^		0.570	1.932		
*P-*value		0.723	0.094	0.689	

Authors' own computation.

### 3.2. Effect of demographic variables on HVLT-R and BVMT-R test scores

#### 3.2.1. Gender

**Figure 1:**
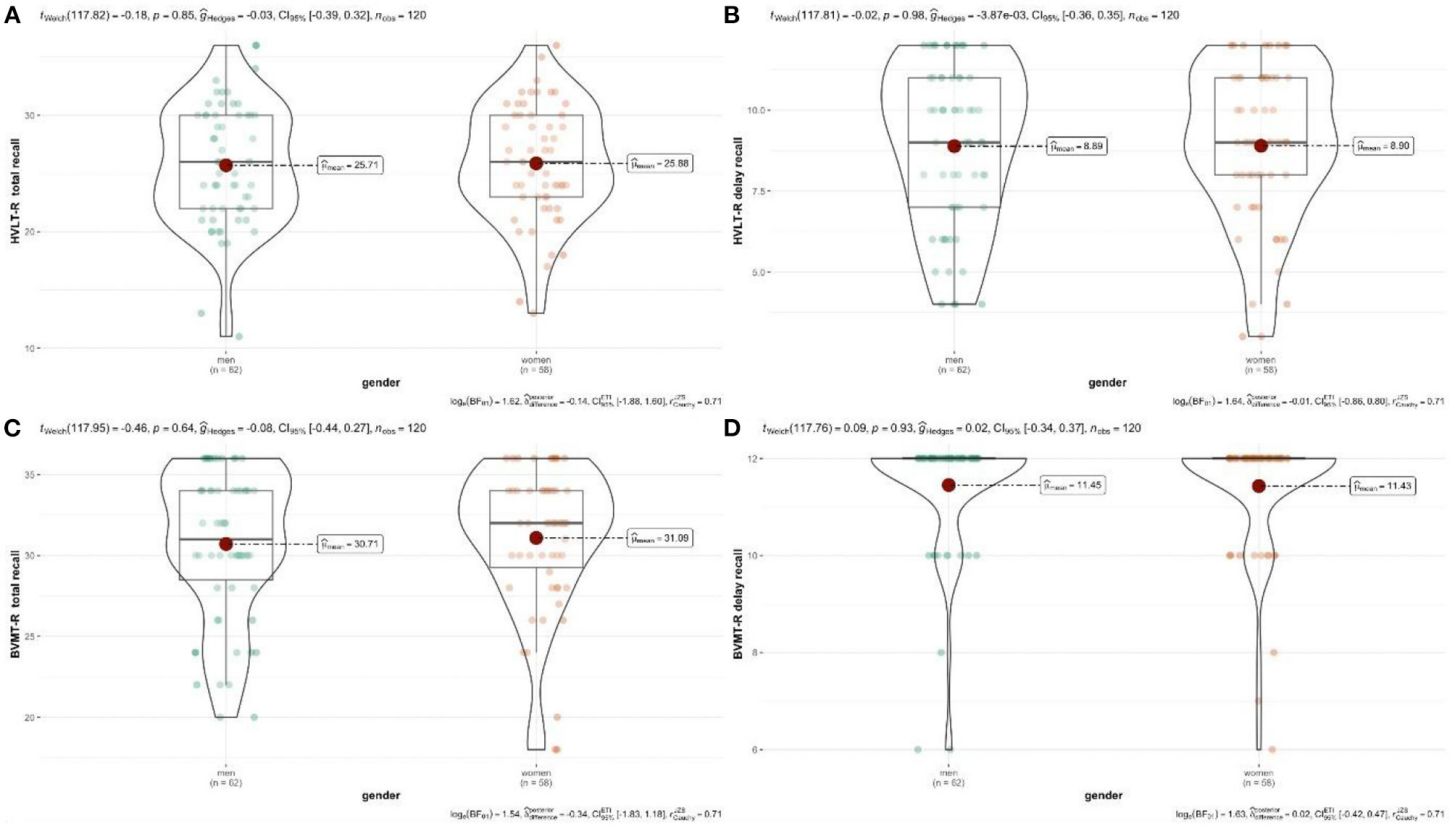
**(A–D)** Relationship between gender and test scores. Authors' own computation.

There was no significant difference in the Total Learning and the Delayed Recall between HVLT-R and BVMT-R at the level of gender, according to the analysis of the generalized linear model (Table 2). As shown in Figure 1, the total number of HVLT-R and BVMT-R learning and the total number of delay did not increase or decrease significantly for male and female.

**Table 2:** Gender comparison of HVLT-R/BVMT-R total learning and total delay scores.

**Item**	**Women (*****n***=**58)**		**Men (*****n***=**62)**		***B***	***P*-value**
	***M***	***SD***	***M***	***SD***		
The total number of HVLT-R learning	25.88	4.97	25.71	5.11	0.0377	0.966
The total number of HVLT-R delays	8.90	2.40	8.89	2.46	−0.012	0.976
The total number of BVMT-R learning	31.09	4.34	30.71	4.55	0.278	0.728
The total number of BVMT-R delays	11.43	1.29	11.45	1.31	−0.060	0.800

Authors' own computation.

#### 3.2.2. Education level

Since the primary education in China lasts for 9 years, the < 10 years group represents that the primary education of the subjects has not been completed. Since the secondary education in China is 3 years, the 10–12 years group represents that the subjects have completed their primary education, but not their secondary education. The >12 years group represents the completion of the subjects' secondary education.

Different levels of education showed different mean levels in cognitive tests (Table 3).

**Table 3:** Education level comparison of HVLT-R/BVMT-R total number of learning and total delay scores.

**Item**	**Education level (year)**	***M***	***SD***
The total number of HVLT-R learning	< 10	24.64	4.42
	10–12	25.36	4.24
	>12	27.04	5.66
The total number of HVLT-R delays	< 10	8.58	2.26
	10–12	8.76	2.47
	>12	9.24	2.54
The total number of BVMT-R learning	< 10	30.18	4.80
	10–12	30.24	4.67
	>12	31.86	3.85
The total number of BVMT-R delays	< 10	11.22	1.61
	10–12	11.44	1.08
	>12	11.64	1.05

Authors' own computation.

**Figure 2:**
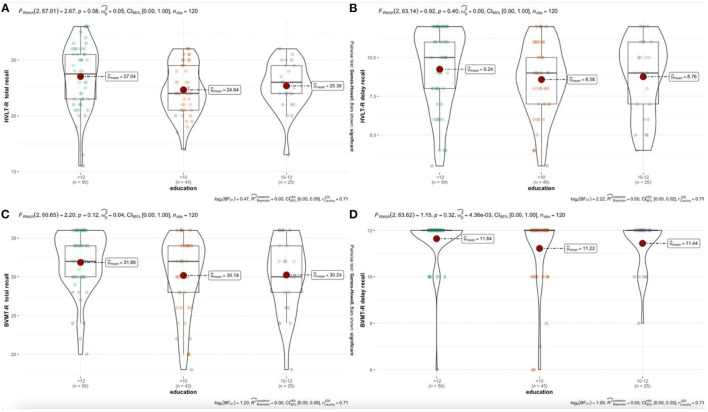
**(A–D)** Relationship between education years and test scores. Authors' own computation.

According to the analysis of the generalized linear model (Table 4), there is no statistical difference among the four test education groups, and there is no linear relationship between education level and test scores. However, as shown in Figure 2, by comparing mean (*m*), standard deviation (*SD*), regression coefficient (*B*), and trend graph analysis, Total Learning and delayed recall of HVLT-R and BVMT-R increased with the increase in education level.

**Table 4:** Relationship between education level and test scores.

**Groups *P*-value**	<**10 years group**		**10–12 years group VS >12 years group**
	**10–12 years group**	>**12 years group**	
The total number of HVLT-R learning	0.562	0.018	0.190
The total number of HVLT-R delays	0.763	0.183	0.436
The total number of BVMT-R learning	0.955	0.063	0.110
The total number of BVMT-R delays	0.499	0.116	0.440

Authors' own computation.

#### 3.2.3. Age

**Figure 3:**
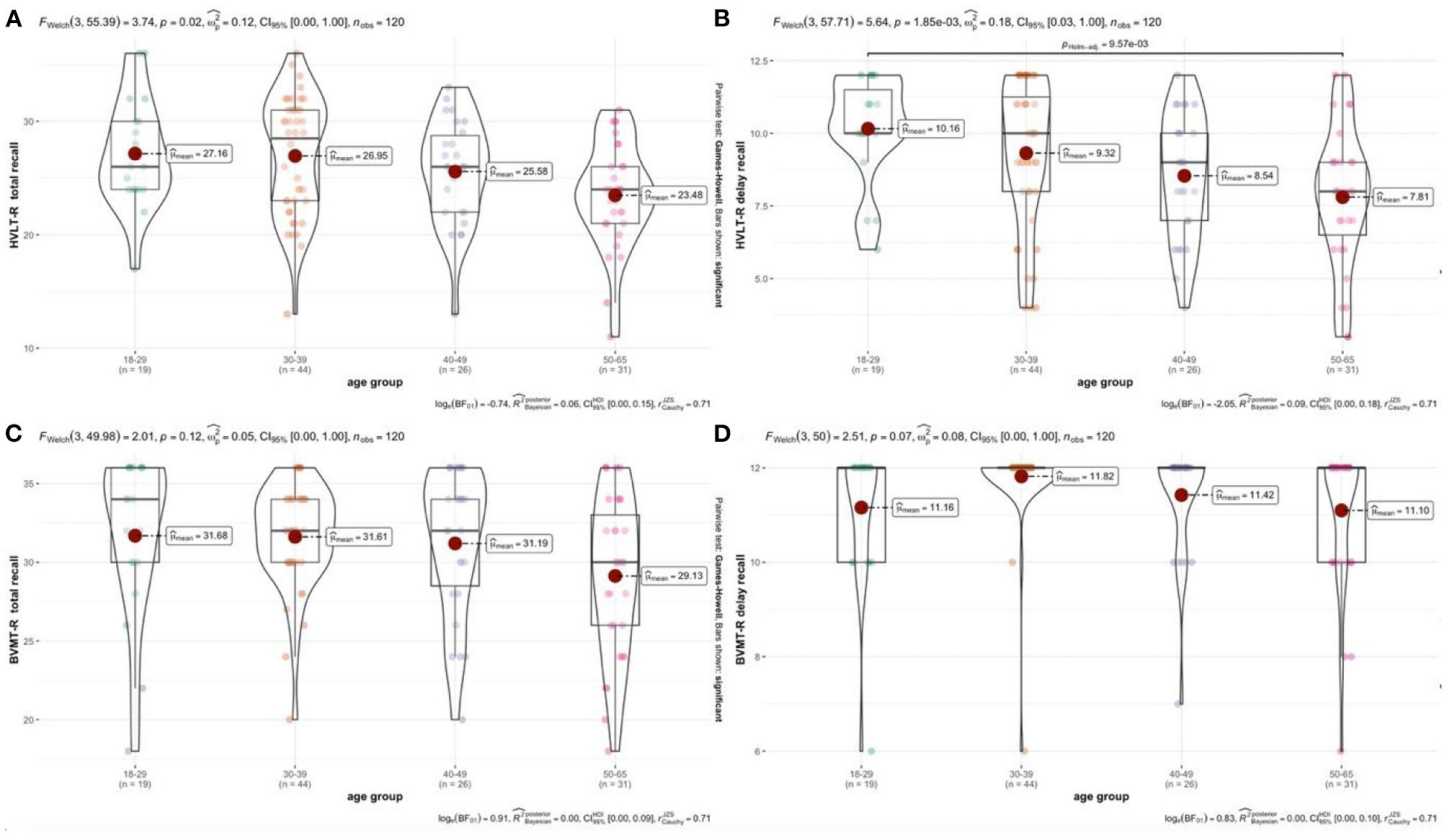
**(A–D)** Relationship between age and test scores. Authors' own computation.

According to the analysis of generalized linear model, the 18–29 age group show the best overall performance. There is no significant difference between the 30–39 age group and the 18–29 age group. However, there are significant differences in the HVLT-R Total Learning and the Delayed Recall between the 18–29, 30–39, and 50–65 age groups. There are significant differences in the four test variables for HVLT-R and BVMT-R between the 30–39 and 50–65 age groups. Different age groups show different mean levels on cognitive tests (Table 5). From Table 6, the four test variables of HVLT-R and BVMT-R are not significant between 40–49 years old and 50–65 years old. Comparing the mean (m), standard deviation (SD), regression coefficient (B), and trend chart analysis, the total number of learning and delay of HVLT-R and BVMT-R decrease with age (Figure 3).

**Table 5:** Comparison of the total number of HVLT-R/BVMT-R learning and total delay of each age group.

**Item**	**Age group (years old)**	***M***	***SD***
The total number of HVLT-R learning	18–29	27.16	4.78
	30–39	26.95	5.18
	40–49	25.58	4.63
	50–65	23.48	4.63
The total number of HVLT-R delays	18–29	10.16	1.80
	30–39	9.32	2.50
	40–49	8.54	2.16
	50–65	7.81	2.41
The total number of BVMT-R learning	18–29	31.68	5.18
	30–39	31.61	3.29
	40–49	31.19	4.56
	50–65	29.13	4.97
The total number of BVMT-R delays	18–29	11.16	1.54
	30–39	11.82	0.95
	40–49	11.42	1.21
	50–65	11.10	1.54

Authors' own computation.

**Table 6:** Relationship between age and test scores.

**Groups *P*-value**	**18–29 years old**		**30–39 years old**		**40–49 years old**	
	**30–39**	**40–49**	**50–65**	**40–49**	**50–65**	**50–65**
The total number of HVLT-R learning	0.879	0.281	0.010^*^	0.254	0.002^**^	0.089
The total number of HVLT-R delays	0.186	0.020^*^	0.000^**^	0.188	0.007^**^	0.232
The total number of BVMT-R learning	0.953	0.709	0.045	0.685	0.012^*^	0.105
The total number of BVMT-R delays	0.059	0.490	0.869	0.190	0.012^*^	0.379

Authors' own computation.

^*^p < 0.05.

^**^p < 0.01.

### 3.3. The scores and differences of HVLT-R and BVMT-R in 120 subjects were compared

Using one-way ANOVA, it is found that there is a significant difference in the total number of HVLT-R (*F* = 2.673, *P* = 0.025). Scores on cognitive tests improve with the learning and me mory process (Table 7). From Table 8, there is no significant difference in the other three tests (*F* = 1.596, *P* = 0.167); the BVMT-R Total Learning (*F* = 1.578, *P* = 0.172); BVMT-R Delayed Recall (*F* = 1.107, *P* = 0.361).

**Table 7:** Scores of 6 versions of HVLT-R and BVMT-R in 120 subjects.

**HVLT-R test**	**1**	**2**	**3**	**The total number of HVLT-R learning**	**The total number of HVLT-R delays**
Mean ± SD	6.79 ± 1.99	9.03 ± 1.99	9.97 ± 1.76	25.79 ± 5.02	8.89 ± 2.42
BVMT-R test	1	2	3	The total number of BVMT-R learning	The total number of BVMT-R delays
Mean ± SD	8.58 ± 2.49	10.85 ± 1.69	11.47 ± 1.18	30.89 ± 4.44	11.44 ± 1.30

Authors' own computation.

**Table 8:** Comparison of differences between groups of HVLT-R and BVMT-R in 120 subjects.

**Item**	***F***	***P-*value**
The total number of HVLT-R learning	2.673	0.025^*^
The total number of HVLT-R delays	1.596	0.167
The total number of BVMT-R learning	1.578	0.172
The total number of BVMT-R delays	1.107	0.361

Authors' own computation. ^*^p < 0.05.

### 3.4. The Scores and differences of HVLT-R and BVMT-R in 20 subjects

#### 3.4.1. The differences between HVLT-R and BVMT-R in 20 subjects were compared

The K-Correlation sample test is used in the non-parametric test to analyze the overall group differences of the 6 versions. Likewise, two correlation sample tests are used to compare afterward. The K-Correlation sample test is used in the non-parametric test to analyze the overall group differences of the 6 versions. Likewise, two correlation sample tests are used to compare afterward. The same group of subjects performed differently in different versions. Similarly, scores on cognitive tests improved over time as learning and memory progressed (Table 9). The results show that there are considerable differences in test scores among different versions (Table 10). The results of the pairwise comparison show that the HVLT-R Delayed Recall is significantly different between form 1 and form 2, 3 and 5 (Table 11). There are noteworthy differences between form 3, form 5, and form 6 (Table 11). The total number of BVMT-R delay in form 2, 3, 5, 6 and form 4, form 1 and form 6 have significant differences (Table 12). There are critical differences in the BVMT-R Total Learning: form 1 and form 3, 5, 6; form 2 and form 4, 5; form 3 and form 4; form 4 and form 5, 6 (Table 12).

**Table 9:** Scores of 6 versions of HVLT-R and BVMT-R in 20 subjects.

**HVLT-R test**	**1**	**2**	**3**	**The total number of HVLT-R learning**	**The total number of HVLT-R delays**
Form1	6.25 ± 2.20	9.00 ± 1.75	9.85 ± 1.66	25.00 ± 5.03	9.65 ± 1.73
Form2	7.20 ± 1.70	9.05 ± 1.93	10.45 ± 1.23	26.70 ± 4.01	10.45 ± 1.23
Form3	8.00 ± 1.86	9.70 ± 1.66	10.50 ± 1.50	28.20 ± 4.49	10.50 ± 1.50
Form4	7.20 ± 2.22	9.20 ± 2.02	10.50 ± 1.24	26.90 ± 4.91	9.90 ± 1.48
Form5	7.85 ± 1.66	9.55 ± 2.16	10.70 ± 1.53	28.10 ± 4.85	10.50 ± 1.57
Form6	7.70 ± 1.87	9.65 ± 1.66	10.05 ± 1.67	27.40 ± 4.72	10.25 ± 1.52
**BVMT-R test**	**1**	**2**	**3**	**The total number of BVMT-R learning**	**The total number of BVMT-R delays**
Form1	7.70 ± 2.54	10.10 ± 2.10	11.00 ± 1.65	28.80 ± 5.85	10.09 ± 1.65
Form2	9.00 ± 2.00	10.60 ± 2.06	11.40 ± 1.47	31.00 ± 4.92	11.40 ± 1.47
Form3	9.30 ± 2.36	11.30 ± 1.87	11.50 ± 1.82	32.10 ± 5.49	11.50 ± 1.82
Form4	8.00 ± 2.03	9.70 ± 2.23	10.15 ± 2.11	27.85 ± 5.62	10.20 ± 1.91
Form5	10.30 ± 2.08	11.00 ± 1.65	11.40 ± 1.47	32.70 ± 4.55	11.40 ± 1.47
Form6	9.80 ± 1.94	10.90 ± 2.00	11.30 ± 1.50	32.00 ± 5.03	11.60 ± 1.39

Authors' own computation.

**Table 10:** Comparison of differences between the 6 versions of HVLT-R and BVMT-R in 20 subjects.

**Item**	**c2**	***P-*value**
The total number of HVLT-R learning	13.523	0.019^*^
The total number of HVLT-R delays	14.097	0.015^*^
The total number of BVMT-R learning	22.354	0.000^**^
The total number of BVMT-R delays	21.490	0.001^**^

Authors' own computation. ^*^p < 0.05, ^**^p < 0.01.

**Table 11:** Total number of HVLT-R delays/learning in 20 subjects.

**Item**	**Forms**	**Forms**	***P-*value**
The total number of HVLT-R delays	Form1	Form2	0.003^**^
	Form1	Form3	0.037^*^
	Form1	Form5	0.013^*^
The total number of HVLT-R learning	Form1	Form3	0.003^**^
	Form1	Form5	0.007^**^
	Form1	Form6	0.005^**^
	Form2	Form5	0.041^*^

Authors' own computation. ^*^p < 0.05, ^**^p < 0.01.

**Table 12:** Total number of BVMT-R delays/learning in 20 subjects.

**Item**	**Forms**	**Forms**	***P*-value**
The total number of BVMT-R delays	Form1	Form6	0.035^*^
	Form2	Form4	0.007^**^
	Form3	Form4	0.015^*^
	Form4	Form5	0.004^**^
	Form4	Form6	0.004^**^
The total number of BVMT-R learning	Form1	Form3	0.013^*^
	Form1	Form5	0.003^**^
	Form1	Form6	0.025^*^
	Form2	Form4	0.018^*^
	Form2	Form5	0.044^*^
	Form3	Form4	0.007^**^
	Form4	Form5	0.002^**^
	Form4	Form6	0.010^**^

Authors' own computation. ^*^p < 0.05, ^**^p < 0.01.

## 4. Discussion

Six versions of HVLT-R and BVMT-R is evaluated for equivalence. We evaluated the equivalence of the six form versions of the HVLT-R and BVMT-R in the Chinese population. In a previous study of the HVLT-R with a similar design, 432 subjects are randomly assigned a version. There is no difference between versions. 18 subjects complete all six versions of the test and take one version for test every 6 weeks. It is recommended that when the HVLT-R is used as a repetition test, forms 1, 2, and 4 are equivalent and slightly more challenging than forms 3, 5, and 6 (29).

It is also investigated how trustworthy the BVMT-R manual copies are. Another study also examined the equivalence of the six versions of the BVMT-R. One test version is given to 600 subjects at random. It is discovered that the BVMT-R groups do not significantly differ from one another. Every week, 18 subjects complete six different versions of the BVMT-R manual. No significant differences are found (28).

Based on the research evidence in the research manuals of BVMT-R and HVLT-R, we assume that the 6 versions of the 2 tests are equivalent to each other. But in this study, the expected results are not quite the same. The 6 versions of HVLT-R and BVMT-R are not completely equivalent. There is a significant difference in the total number of HVLT-R between the 6 versions of 120 subjects (*F* = 2.673, *P* = 0.025). No differences are found in the remaining three indicators, including the HVLT-R Delayed Recall (*F* = 1.596, *P* = 0.167), the BVMT-R Total Learning (*F* = 1.578, *P* = 0.172) and BVMT-R Delayed Recall (*F* = 1.107, *P* = 0.361). There are differences in four indexes among the six versions of 20 subjects, including the HVLT-R Total Learning (χ2= 13.523, *P* = 0.019), the HVLT-R Delayed Recall (χ2= 14.097, *P* = 0.015), the total number of BVMT-R studies (χ2= 22.354, *P* = 0.000) and the BVMT-R Delayed Recall (χ2= 21.490, *P* = 0.001).

The HVLT-R test do not only evaluate short-term memory skills. At the same time, some test takers struggle to identify the rules of semantic classification in the initial exam, which result in a poor Form1 score. Additionally, the number of words that could be remembered depends on how familiar they are with the words in each version, such as fork and sweet wine in Form2 or canary, uniform, robin, driver, chisel, and other uncommon words in Form4. As a result, the scores of different versions are different. For the BVMT-R test, there are only 10 s to observe the figure time, which is exceptionally sensitive to the instantaneous fluctuation of attention. With the understanding of the test and the increase in concentration, the degree of completion gradually improves, which could account for the low score of Form1. At the same time, the completion of the graph might also be affected by the similarity of the graph. The subjects are unable to provide consistent responses to the questions. In addition, we find that the degree of completion of irregular closed and open graphics in Form2 and Form4 is not high compared with other versions, which is the main reason why we consider the low score of versions. The current research results show that not all versions are equivalent. Versions 1, 2, and 4 of the HVLT-R and BVMT-R were rather difficult, while versions 3, 5, and 6 are relatively simple. The results are more reliable because 20 subjects who complete all 6 sets of tests are more sensitive to the difference in difficulty between versions than 120 subjects who complete one of the 6 sets of tests. We could use it as a very beneficial reference when using the two tests in China.

Age, education and gender usually affect the performance of neuropsychological tests. Although norm data can correct these variables in many tests, little research has been done on the Hopkins Verbal Learning Test-Revised and the short visual-spatial memory test. The HVLT-R manual does not include gender-specific norm data and provides a more detailed description of the specification sample. Among them, women aged 16–92 (75.2%) are the most unbalanced. The gender distribution is most unbalanced among older adults, particularly between the ages of 70 and 79, where 90% of the population consists of women. The HVLT-R manual describes the results of a stepwise multiple regression, which shows that gender has little effect on the variance of all learning and memory scores, but it is statistically significant, accounting for 1.7% of the variance of learning scores. It accounts for 1.4% of the variance of delayed recall scores. Age is responsible for 18.8% of the variance in learning scores, 12.2% of the variance of delayed recall scores. Education level is responsible for 5.1% of the variance of learning scores, and 3.3% of the variance of delayed recall scores. In their standard sample, gender is a significant determinant of the HVLT-R learning and memory, but in comparison to other demographic factors, it has no clinical significance. The common standard is implemented along with the BVMT-R manual's design, which is similar to that of the HVLT-R. Separate norms for men and women are not included in the measure either. In terms of age, the BVMT-R standard sample is rather comparable to the U.S. Census, but it does not offer any descriptive statistics to indicate the distribution of men and women in the sample. After accounting for age, it is stated in the BVMT-R manual. Per the BVMT-R manual, after accounting for age, gender and education do not appear to have much of an impact on test results.

Vanderploeg et al. (30) provided age and gender corrections for the HVLT-R test Form1 in a sample of 394 elderly participants. Consistent with other studies, age is negatively correlated with scores on measures of learning and memory, with where women score higher than men. The authors also examine the impact of education and conclude that it has no appreciable influence on variations in HVLT-R scores. The study still has some flaws despite the fact that the data suggest some new demographic effects. For instance, the sample selection cannot accurately reflect the population as a whole, and the inclusion criteria for these subjects are vague. Low total recall scores are found in the study. Additionally, the delayed recall test includes cues, which could impact the results of the recognition test.

The age and health of 172 elderly people are investigated by Gale et al. As expected, the older the age, the lower the BVMT-R score. And, women tend to perform slightly better than men. These test results appear to be unaffected by education. Although the BVMT-R score in an older cohort is revised in this study, it may be useful. But it has some glaring drawbacks. For instance, only three BVMT-R scores (total score of type-3 learning, delayed recall, and recognition) are modified in this study, whereas clinicians could benefit from the full range of BVMT-R scores. This study only used the BVMT-R test Form4 (e.g., single learning test, retention percentage, false positivity). Low recall scores are also revealed by the study (31). Two other studies report demographic corrections for HVLT-R and BVMT-R (32, 33). But these are in the non-elderly cohort (e.g., 20–65 years old).

The demographic data of this study suggest that: the analysis of the generalized linear model reveals that there is no significant difference in the influence of gender effect across the four tests. In general, men have a better spatial memory than women, while women have a better verbal memory. This study has limitations, regardless of the fact that the difference is not statistically significant. Only the HVLT-R and BVMT scores for learning style and delayed recall are investigated. There was neither a test of recognition nor a percentage of retention.

There was no significant difference in the effect of education level among the four tests. By comparing mean (*m*), standard deviation (*SD*), regression coefficient (*B*), and trend graph analysis, the total number of learning and delay of HVLT-R and BVMT-R has an upward trend with the increase in education level. BVMT-R test is related to the speed of information processing. At the same time, the figure is a cross-cultural and cross-educational level test and reflects the underlying level of neurocognition. It is related to the degree of congenital neural development, and may not have much to do with acquired learning. We conclude that the difference in BVMT-R is statistically insignificant. The HVLT-R test contains relatively simple vocabulary. In China, everyone is required to attend school for 9 years. Therefore, there is no discernible difference in the familiarity with the word list between those with a 9-year education and those with a higher level of education. We believe this is why there is no significant difference in the impact of education level on the HVLT-R test. Because of the small sample size, additional research is required.

In terms of age effect, by comparing the mean (*m*), standard deviation (*SD*), regression coefficient (*B*), and trend chart analysis, the total number of learning and delay of HVLT-R and BVMT-R decrease with the increase of age. The 18–29-year-old group show the best performance, while the 18–29-year-old group has no significant difference with the 30–39-year-old group, and decrease significantly after the 30–39-year-old group. It shows that the memory in the human youth reaches a certain peak, showing a gradual downward trend, and the change is not obvious after middle age. For elderly people over 65 years old, this study has not been included. Whether there will be further decline with aging needs further research.

Research advantages: by providing more options when repeat tests are required, the validation of test version equivalence reduces the exercise effect. The results from this study could be used as a guide for upcoming studies and clinical applications in China.

This study has the following limitations:

(1) The age span is limited to 18–65, without involving children, adolescents and the elderly. And its application in this kind of population needs to be further verified.
(2) The choice of test interval will also affect the stability of the results. Every week, 20 subjects complete six different versions of the BVMT-R and HVLT-R manual. Subjects with a short time interval and better long-term memory may record the words or figures they answered last time in the answer book, causing confusion and failing to get the corresponding score.
(3) There is a lack of delayed recognition test. Due to the limitation of time and energy, this study only conducted learning and delay tests according to the settings in MCCB.

For future research, it can be added to the test of recognition and the consistency between raters. In the majority of clinical and research applications. We need frequency evaluate cognitive improvement in clinical settings. In order to avoid the effect of practice, we typically choose to change the version. when the difference is small, it is impossible to determine whether it is the result of a genuine clinical intervention or a slight difference following the version change. Our future research also needs to take into account: defining criteria for giving partial scores, ensuring that subjects understand the task, standardized instructions, strict time control, and not passing on any information that might influence the response. These can be used to ensure the quality of the test. Delayed recognition tests should be added in future studies. In the future, these issues can be investigated further in terms of statistics and methodology.

## Data availability statement

The original contributions presented in the study are included in the article/supplementary material, further inquiries can be directed to the corresponding authors.

## Ethics statement

The majority of the information created or analyzed during this study is presented and discussed in this article. The Peking University Institute of Mental Health's Ethics Committee reviewed and gave its approval to all studies involving human subjects. The patients/participants provided their written informed consent to participate in this study.

## Author contributions

CS and XH conceptualized the article. CS, GC, TY, and XY reviewed articles and collected data. TY ran the analysis. YC formulated the initial version of the article and revised it based on co-authors' comment threads. The article was reviewed and approved by all authors. All authors contributed to the article and approved the submitted version.
